# Neuron navigator 2 is a novel mediator of rheumatoid arthritis

**DOI:** 10.1038/s41423-021-00696-7

**Published:** 2021-07-28

**Authors:** Ran Wang, Meng Li, Qian Ding, Jianghong Cai, Yue Yu, Xinhua Liu, Jianchun Mao, Yi Zhun Zhu

**Affiliations:** 1 State Key Laboratory of Quality Research in Chinese Medicine, School of Pharmacy, Macau University of Science and Technology, Avenida Wai Long, Taipa, Macau China; 2 Shanghai Key Laboratory of Bioactive Small Molecules, Department of Pharmacology, School of Pharmacy, Fudan University, Shanghai, China; 3 Department of Rheumatology, Longhua Hospital of Shanghai University of Traditional Chinese Medicine, Shanghai, 200030 China

**Keywords:** Chronic inflammation, Mechanisms of disease

Rheumatoid arthritis (RA) is a systemic autoimmune disease mainly characterized by synovial tumor-like hyperplasia, synovial inflammation, and damage to adjacent cartilage and bones.^1^ Our previous study provided evidence that cystathionine-γ-lyase (CSE) and the histone demethylase JMJD3 are significantly increased in RA, and CSE was found to negatively regulate the inflammatory response in fibroblast-like synoviocytes (FLSs), which was mainly attributed to JMJD3 inhibition, in RA.^2^ Our recent data provided further novel insight into the molecular mechanism by which the inflammatory response is regulated in RA pathogenesis. As a member of the neuron navigator (NAV) family, NAV2 was reported to be associated with deeper invasion and lymph node and distant metastases in colorectal carcinoma.^3^ Our latest study provides evidence that NAV2 expression is clearly higher in RA patients. TNF-α intervention can significantly increase the level of NAV2, promote proliferation and invasion and further trigger the Wnt/β-catenin signaling pathway. Neutralization or elimination of NAV2 expression may significantly ameliorate RA severity or suppress the development of autoimmune disorders.^4^

During the progression of RA, TNF-α is an important physiological inflammatory mediator that exerts a variety of biological effects by acting on different receptors on target cells.^5^ TNF-α plays a key role in the pathogenesis of RA and maintenance of chronic synovial inflammation in joints.^6^ Therefore, in our study, we used TNF-α to stimulate MH7A FLSs to study proliferation and invasion in RA.

In our study, we reveal that NAV2, a member of the neuron navigator family, plays a very important role in the occurrence and development of inflammation in RA by activating the Wnt/β-catenin signaling pathway. NAV2 was first identified as an all-*trans* retinoic acid (atRA)-responsive gene in humans that is essential for normal development of the vertebrate nervous system.^7^ A previous study also clearly showed that NAV2 is overexpressed in human colorectal cancer (CRC) and plays a key role in promoting CRC cell invasion and metastasis by regulating F-actin polymerization via the SSH1L/Cofilin-1 pathway.^8^ However, the function of NAV2 in RA and relationship between NAV2 and RA pathogenesis remain unclear. Because invasion and migration can also occur in RA, we hypothesized that NAV2 regulates proliferation and invasion to promote the progression of RA. First, we verified the presence of increased NAV2 levels in RA patients. We also demonstrated NAV2 protein overexpression in synovial tissues in rats with adjuvant-induced arthritis (AIA) compared with normal rats and in TNF-α-induced MH7A cells compared with control MH7A cells. We further confirmed that elevated NAV2 expression promoted MH7A FLS proliferation and invasion. Importantly, knockdown of NAV2 by siRNA inhibited NAV2-induced production of the inflammatory mediators MMP-9, COX-2, and IL-6 in vitro, indicating that NAV2 plays a crucial role in the inflammatory response by regulating the expression of inflammatory mediators in RA.

The Wnt/β-catenin signaling pathway is a key pathway involved in cellular proliferation, cell migration, and tissue homeostasis and regeneration, as well as embryonic development.^9^ Furthermore, dysregulation of the Wnt/β-catenin signaling pathway is believed to be involved in the pathogenesis of cancer, vascular disorders, and autoimmune diseases.^10^ RA is closely related to abnormal activation of the Wnt/β-catenin signaling pathway, which participates in multiple pathological processes, such as maintenance, proliferation, differentiation and self-renewal.^11^ Another study reported that NAV2 might facilitate epithelial-to-mesenchymal transformation (EMT) in melanoma cells by activating the GSK-3β/β-catenin signaling pathway.^3^ Based on the above findings, we wondered whether NAV2 could modulate the Wnt/β-catenin signaling pathway to promote the proliferation and invasion of FLSs in RA. Herein, we found that the expression of GSK-3β, β-catenin, c-myc and CyclinD1 and the downstream proteins MMP-3 and MMP-9, proteins in the MMP family that are closely related to cell migration and invasion, was dramatically increased in AIA rats and TNF-α-induced MH7A cells. Knockdown of NAV2 in MH7A cells by siRNA dramatically impaired the expression of proteins in the Wnt/β-catenin signaling pathway and reversed the phenotypes observed upon TNF-α stimulation. Taken together, these data suggest that the Wnt/β-catenin signaling pathway could be activated by NAV2 in MH7A cells in vitro to further promote the progression of RA.

**Fig. 1:**
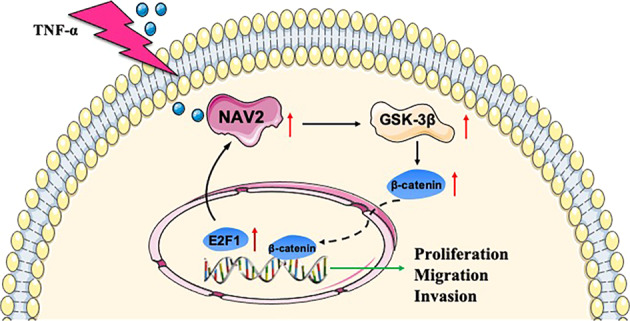
A model of the possible mechanism by which NAV2 mediates the inflammatory response in TNF-α-induced MH7A cells

Furthermore, to decipher the underlying regulatory mechanism of NAV2 in RA, we used the PROMO and JASPAR databases to predict the transcription factors involved in the regulation of NAV2 and found that the transcription factor E2F1 promotes NAV2 promoter activity and activates NAV2 transcription. Moreover, a ChIP assay also verified that E2F1 specifically binds the NAV2 promoter region (Fig. 1).

In conclusion, our present study reveals an important novel finding: NAV2 plays a critical role in RA. Furthermore, we provide a new understanding of the molecular mechanism of RA, which is unique and previously unreported. We also speculate that targeting NAV2 might not only affect inflammation in RA but also interfere with a major type of cell-cell interaction involved in the sensitization of joint-innervating neurons, which drive pain in arthritis.^12^ Therefore, NAV2 provides an attractive novel target for intervention in inflammatory diseases, especially RA, and we are quite eager to learn more.
